# Electronic Tunability and Cancellation of Serial Losses in Wire Coils

**DOI:** 10.3390/s22197373

**Published:** 2022-09-28

**Authors:** Roman Sotner, Jan Jerabek, Ladislav Polak, Radek Theumer, Lukas Langhammer

**Affiliations:** 1 Department of Radio Electronics, Faculty of Electrical Engineering and Communication, Brno University of Technology, Technicka 3082/12, 616 00 Brno, Czech Republic; 2 Department of Telecommunications, Faculty of Electrical Engineering and Communication, Brno University of Technology, Technicka 3082/12, 616 00 Brno, Czech Republic

**Keywords:** active filter, current conveyor, electronic adjustment, inductance simulator, loss cancellation, oscillator, variable gain amplifier

## Abstract

This work presents a novel methodology to adjust the inductance of real coils (electronically) and to cancel out serial losses (up to tens or even hundreds of Ohms in practice) electronically. This is important in various fields of electromagnetic sensors (inductive sensors), energy harvesting, measurement and especially in the instrumentation of various devices. State-of-the-art methods do not solve the problem of cancellation of real serial resistance, which is the most important parasitic feature in low- and middle-frequency bands. In this case, the employment of serial negative resistance is not possible due to stability issues. To solve this issue, two solutions allowing the cancellation of serial resistance by the value of the passive element and an electronically adjustable parameter are introduced. The operational ranges are between 0.1 and 1 mH and 0.1 and 10 mH, valid in bandwidths from hundreds of Hz up to hundreds of kHz. The proposed concepts are experimentally tested in two applications: an electronically tunable oscillator of LC type and an electronically tunable band-pass RLC filter. The presented methodology offers significant improvements in the process of circuit design employing inductors and can be beneficially used for on-chip design, where serial resistance issues can be very significant.

## 1. Introduction

Many electronically adjustable applications, designed for various frequency bands, expect the utilization of active elements (AE) [1,2] in the implementation of circuits employing only resistors and capacitors as inertial passive elements. The behavior of the inductor is frequently obtained from a capacitor-based impedance response, among others by so-called impedance inverters [3] and gyrators [4] (also known as inductance simulators). These devices allow the adjustment of the multiplication or conversion constant; thus, they also ensure electronic adjustability. They are known as impedance multipliers, intended especially for large-range variation in capacitance values (see, for example, [5,6,7,8,9,10] and references cited therein) or inductance values converted from capacitance values (examples available in [11,12,13,14,15,16,17,18,19,20,21,22,23,24] and references cited therein). These impedance multipliers and converters are very popular for so-called fractional-order designs [25].

Research on impedance multiplication and conversion circuits started more than 50 years ago with basic Antoniou solutions [26,27] using operational amplifiers (OAs) [1,2]. The best known examples [28] of these circuits use operational transconductance amplifiers (OTAs) [1,28] because electronic adjustment of their transconductance (*g*_m_) for tunability purposes is straightforward. These approaches are very useful, especially when the simulated “active” inductance [29] reaches very high or extreme values. Standard passive wired coils with high values of inductance (tens of mH and more) are extremely large (bulky) and heavy devices. Real low-frequency wire coils with values of tens–hundreds of mH (intended for frequencies below hundreds of kHz and for audio bands) are very impractical for up-to-date flexible and compact designs. This was the reason for the development of impedance inverters and converters [26,27,28,29]. However, the load capabilities of these active inductors are limited (impossible to drive large values of currents) and the application of synthetic inductance elements (based on active devices) suffers from the significant impacts of real parasitic features originating from the used AEs (limited bandwidth and nodal parasitic impedances especially).

**Figure 1:**
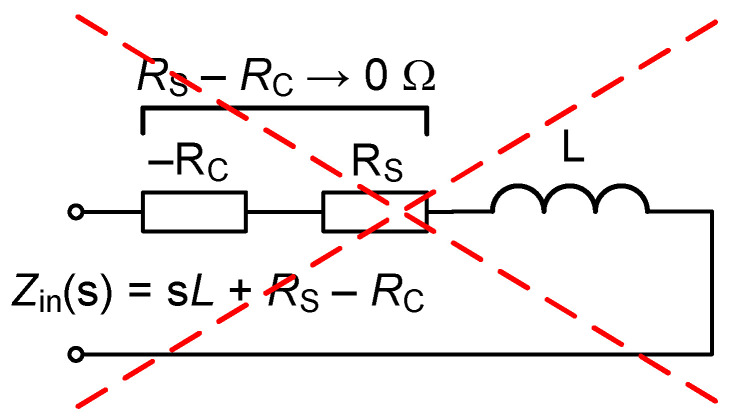
The easiest method for the cancellation of serial resistance of the inductor, inapplicable in linear applications.

**Figure 2:**
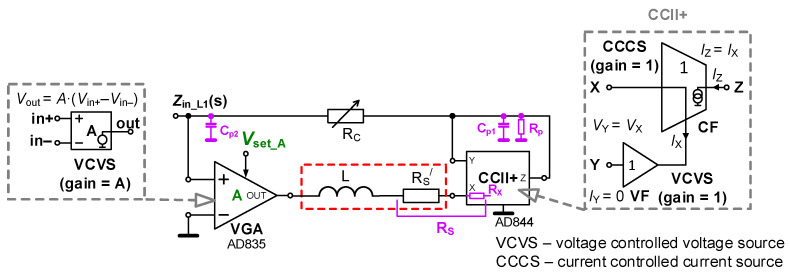
Topology of electronically adjustable inductance using VGA and CCII+ suitable for *R*_S_ cancellation.

**Figure 3:**
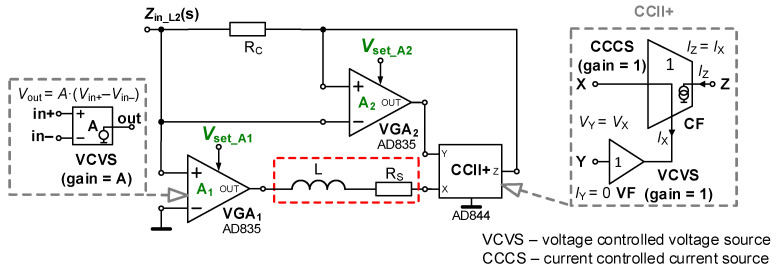
Topology of electronically adjustable inductance value and *R*_S_ cancellation using two VGAs and CCII+.

Nowadays, progress in fabrication technologies (due to the employment of specific materials and layers) brings very small elements (inductors having values in hundreds of μH, units of mH and more) also suitable for low-frequency design (below MHz). Therefore, the final devices are significantly smaller (typically comparable to a package of standard 0.5 W or smaller resistors fabricated in through-hole assembling technology) than wire-based bulky and heavy coils. Unfortunately, standardization of the usage of real inductors in common practice in electronically adjustable systems has several issues, namely (a) the absence of the electronic adjustability of inductance values, (b) the real serial resistance of fabricated inductors (the full inductance modeling involves also other parasitic elements [3], but serial resistance (*R*_S_ in Figure 1, Figure 2 and Figure 3) is the most significant issue [14,22]) and (c) if active inductors based on impedance simulators (converters) are used, there is an identical problem with serial resistance [11,12,13,14,15,16,17,18,19,20,21,22,23,24,25].

A precise comparison of the state-of-the-art (SoA) solutions of electronically controllable inductance simulators is provided in Table 1. The following conclusions can be made: (a) only a limited number of complex solutions (example in [17]) allow the cancellation (neither electronic cancellation) of serial losses, and (b) voltage adjustment of the value of inductance is not a standard feature, except in [17,18,25] (bias DC current used for the remaining solutions is accompanied by some issues—mostly the dependence of terminal resistances on this feature [28]). As is evident, the implementation of active impedance inverter-based inductance simulators allowing the adjustment of the serial resistance value (or its cancellation) brings significant complexity [17].

To the best of the authors’ knowledge, works solving the issue of the serial resistances of real inductors (excluding the inductance simulators presented in Table 1) are not presented in the current SoA. However, this work shows that standard inductance (as a passive element) can be beneficially used in tunable applications, and newly proposed solutions reveal that the serial resistance *R*_S_ of an inductor can be easily cancelled without adding other complex circuitry. The utilization of negative resistance in series with a lossy element (see Figure 1) for cancellation purposes is not possible due to the instability of this solution [30]. These solutions can only be used for border of stability adjustment in oscillators but definitely not in linear applications, where the positive feedback path of the amplifier causes nonlinear comparator operation. A similar issue may occur in negative inductance simulators (see [13,19], for instance) in the case of linear applications.

This paper targets the development of simple active circuitries allowing (a) electronic adjustment of the value of inductance by DC control voltage, (b) the possibility to adjust or eliminate serial losses (serial resistance), (c) stable operation in linear applications and (d) the application of commercially available active devices only for widespread accessibility.

The rest of the paper is organized as follows. Section 2 discusses two novel circuit solutions of adjustable inductance and explains their operation. Section 3 presents the basic features of specific design examples from simulations and lab measurement. In Section 4, two beneficial examples of the proposed solutions (electronically adjustable LC oscillator and LC band-pass filters) are shown. Section 5 summarizes the importance of the proposed methodology and the obtained results for the field of sensing, instrumentation and measurement. Section 6 concludes this paper.

## 2. Circuits Suitable for Inductance Adjustment

### 2.1. Cancellation of Serial Resistance by Passive Element

A variable gain amplifier (VGA) [1] is an example of a device useful for the implementation of the active part of the electronically adjustable impedance [31]. The VGA has a very simple (ideal) principle described by the following equation: *V*_OUT_ = (*V*_+_ − *V*_−_)·*A*, where *A* represents the electronically settable voltage gain.

The presence of the lossy element in the case of inductance may result in a limited value of attenuation in the stop bands of frequency filters, as typical applications of LC elements. Therefore, a method for the elimination of this effect would be very useful. The circuit solution with serial resistance cancellation as well as electronic adjustability of the inductance value is shown in Figure 2. The topology uses a VGA, a current conveyor of second generation (CCII+) [1,2] and one passive element. The outer behavior of the CCII+ device is described by ideal inter-terminal relations, *V*_X_ = *V*_Y_, *V*_Y_ = 0, *I*_Z_ = *I*_X_. The electronically adjustable parameter (*A*) of the VGA serves for the electronic adjustment of the resulting inductance value. Resistor *R*_C_ represents the counterbalance of the external serial resistance of coil *R*_S_^/^ and the parasitic serial resistance of the current input terminal X (*R*_X_). The form of this adjustable impedance can be found as

(1) $$Z_{\mathrm{in}\_L1}(s)=\frac{sL+R_{S}-R_{C}}{A-1}.$$

Supposing the equivalence of *R*_S_^/^ + *R*_X_ = *R*_S_ = *R*_C_, the lossy part disappears and (1) reduces to the form

(2) $$Z_{\mathrm{in}\_L1}(s)=\frac{sL}{A-1},$$

offering tunable inductance *L*_tun_ = *L*/(*A* − 1). The value of voltage gain *A* > 1 should be set for stable operation. Then, this parameter ensures the inversely proportional adjustment of tunable inductance value *L*_tun_.

The relative sensitivities of the *L*_tun_ value to parameters *L* and *A* reach *S*_R_Ltun_^L^ = ∂*L*_tun_/∂*L* × *L*/*L*_tun_ = *L* and *S*_R_Ltun_^A^ = ∂*L*_tun_/∂*A* × *A*/*L*_tun_ = *−A/(A −* 1*).* The purpose of the parasitic elements, marked by magenta color in Figure 2, will be explained later.

### 2.2. Electronic Cancellation of Serial Resistance and Electronic Control of Inductance Value

The full electronic adjustability (inductance value and cancellation of serial losses) is achieved in the topology presented in Figure 3. The resulting form of ideal input impedance yields

(3) $$Z_{\mathrm{in}\_L2}(s)=\frac{sL+R_{S}-R_{C}A_{2}}{A_{1}}.$$

Impedance *Z*_in_L2_ passes into lossless form when *A*_2_ = *R*_S_/*R*_C_, which results in *L*_tun_ = *L*/*A*_1_, where *A*_1_ serves for indirectly proportional adjustment. Compared to the previous case, the denominator has a simpler and more advantageous form. The relative sensitivity of the *L*_tun_ value to parameters *L* and *A*_1_ reaches +1 and −1, respectively.

## 3. Simulation and Experimental Results

The features of the proposed concepts were verified by simulations and also laboratory measurements. In the case of simulation, the PSpice simulation tool using standard macromodels was used. In the case of laboratory measurements, integrated device AD844 (CCII+ part) [32] and voltage multiplier AD835 (VGA) [33] were employed as active elements. The power supply voltage was ±5 V.

The selected method of verification (using the off-the-shelf active elements) brings the fast and simple reproducibility of the presented research for a wide research community. Moreover, the values of real inductances implemented in our design are not available in the process design kit libraries of standard CMOS processes. Therefore, the whole circuit including passive elements cannot be integrated. The use of large values of inductances (μH, mH) is intentional because commercially available, discrete inductances of these values have significant issues with the described serial losses. The presented methodology solves this issue (i.e., values of mH and bands of kHz were selected intentionally for verification, experiments and applications). Moreover, it adds the electronic adjustment of the inductance value. This is important for some practical cases of low-frequency (<kHz) measurement and sensing applications. The low-power CMOS design of the topology is possible but there are also limitations (high-value inductances will be connected externally, decrease in signal voltage levels/dynamics of application, as well as allowed adjustability ranges, compromises in the design defined by values of real parts of impedances of high-impedance nodes).

### 3.1. Cancellation of Serial Resistance by Passive Element—Experimental Verification

**Figure 4:**
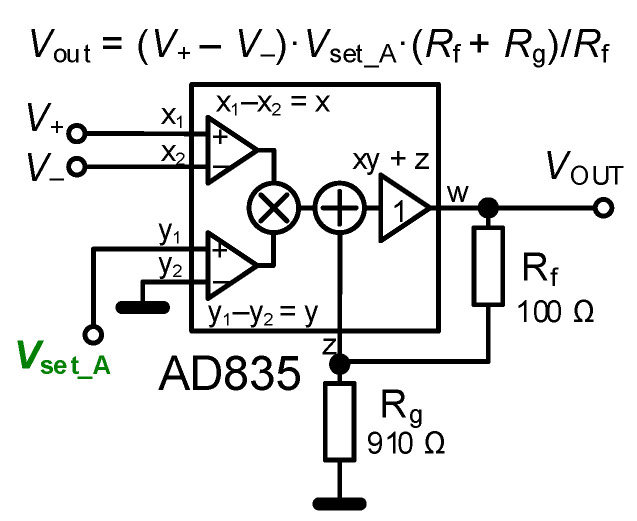
Principle of VGA operation and gain setting.

**Figure 5:**
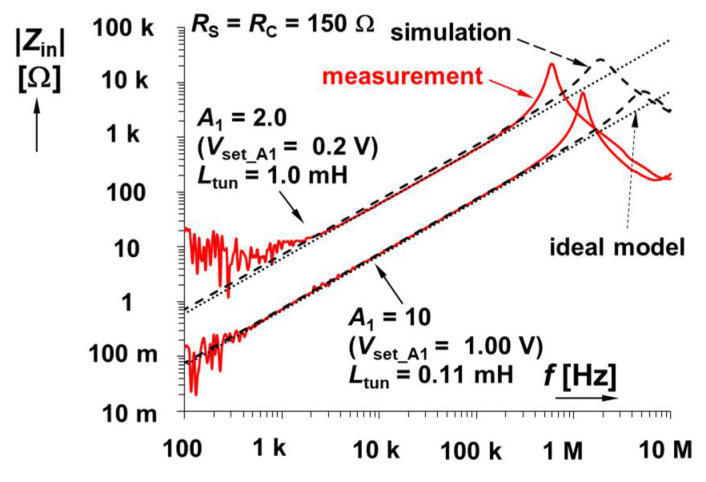
Magnitude impedance plots of solution presented in Figure 2.

Firstly, the topology introduced in Figure 2 was tested. The design parameters and values of elements suppose operation up to hundreds of kHz with an inductance value in the range of 0.1 and 1 mH (*L*_tun_). Next, a real coil with *L* = 1 mH, *R*_S_^/^ = 100 Ω was used. We also have to include the significant parasitic resistance of the X terminal of CCII+ 50 Ω [32] in calculations; therefore, *R*_S_ = 150 Ω was considered. Trimmer *R*_C_ (see Figure 2) with a value of 1.5 kΩ was used as a serial resistance cancellation tool. The value of *R*_C_ was approximately set to *R*_S_ (150 Ω). The trimmer can be set from 0 to 1.5 kΩ. We need to compensate/cancel the value of *R*_S_ ≅ 150 Ω with it. Therefore, the value of *R*_C_ has been set to 150 Ω too. However, we have to consider also the internal resistance of the AD844 X terminal [32] that may suffer from large fabrication tolerance. If it is determined as 50 Ω, for instance, *R*_C_ ≅ 100 Ω. The range of *L*_tun_ adjustment leads to the values of *A* between 10 (0.1 mH) and 2 (1 mH). The driving voltage is set in accordance with the principle shown in Figure 4 to 1 V (0.1 mH) and 0.2 V (1 mH). The results of tunability are presented in the form of magnitude impedance plots (see Figure 5). For this measurement, the Keysight DSO-X 3024T oscilloscope, having the option of Frequency Response Analysis (FRA), and a simple impedance measuring readout were used. The simulation- and measurement-based results indicate *L*_tun_ = 0.12 and 1.13 mH (evaluated at 30 kHz) and *L*_tun_ = 0.12 and 0.95 mH, respectively. The difference between experiment and theory achieves maximally 5% error.

The most affecting small-signal parasitic features are concentrated to high-impedance nodes (see passive elements highlighted in magenta color in Figure 2). These analyses were performed with the help of the simplification mode of symbolical solver SNAP [34]. The visible resonant frequency peak is defined by both parasitic capacities of high-impedance nodes *C*_p1_ and *C*_p2_:

(4) $$\omega_{r}≅\sqrt{\frac{A-1}{L\left(C_{p1}+C_{p2}\right)}},$$

thus, this value varies with *A*. Considering known values from previous discussion (*L* = 0.1 mH, *L* = 1 mH) and small-signal parameters from the datasheet (*C*_p1_ = 2 pF, *C*_p2_ = 7 pF, *R*_p_ = 3–4 MΩ) [32,33], the expected values are *f*_r1_ ≅ 5.04 MHz (*A* = 10) and *f*_r2_ ≅ 1.68 MHz (*A* = 2). These are very close to the results obtained from simulations (see Figure 5). A significant increase in the values of *C*_p1,2_ must be considered in the experimental setup because of the impact of the printed circuit board (tested device + impedance measuring readout) that increases these values by approximately 20 pF (thus, *C*_p1_ = 22 pF, *C*_p2_ = 29 pF). This results in *f*_r1_ ≅ 2.12 MHz (*A* = 10) and *f*_r2_ ≅ 0.71 MHz (*A* = 2), again close to the results of experiments. The minimal value of impedance at the lowest frequencies has the following limit:

(5) $$\left|Z_{\mathrm{in}\_\min}\right|≅\frac{R_{S}R_{C}+R_{S}R_{p}-R_{C}R_{p}}{R_{p}\left(A-1\right)}.$$

It yields |*Z*_in_min_| below tenths of Ω for *A* = 2 → 10 at 0 Hz (DC). These values are sufficiently low so as not to exert undesired influences on the overall performance of the circuit in Figure 2. Experimental results at low frequencies (100 Hz) are significantly influenced by the limits of the measurement methodology and the dynamical limits of the measuring equipment.

### 3.2. Electronic Cancellation of Serial Resistance and Electronic Control of Inductance Value—Experimental Verification

**Figure 6:**
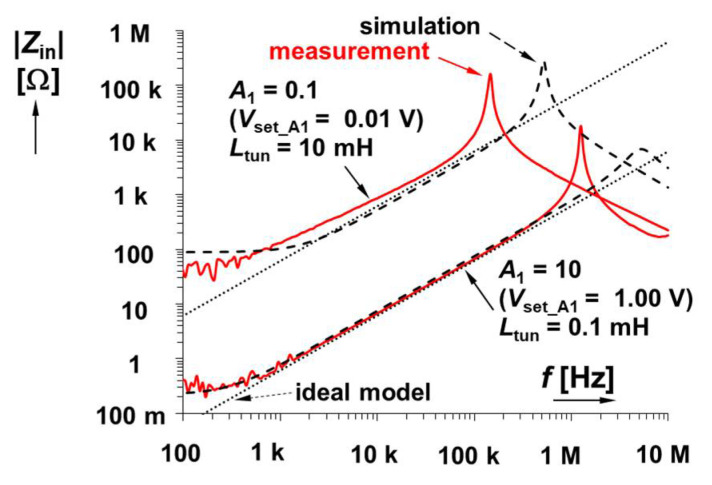
Magnitude impedance plots of solution presented in Figure 3.

**Figure 7:**
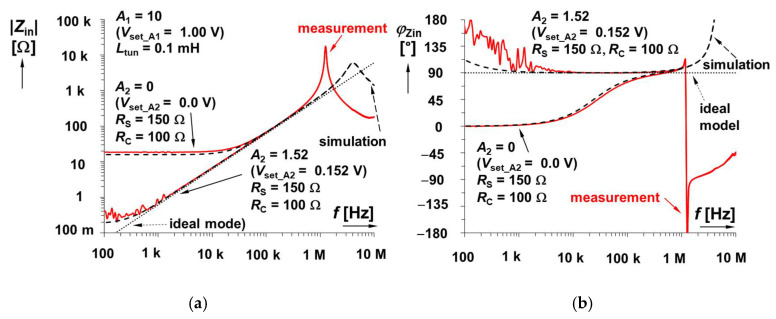
Comparison of lossless and lossy impedance plots for fully electronically adjustable solution in Figure 3: (**a**) magnitudes and (**b**) phases.

Results for the second solution, shown in Figure 3 (a fully electronically adjustable circuit), are plotted in Figure 6. For its analysis, the value of *R*_C_ was set to 100 Ω (*R*_S_ is still 150 Ω) because direct equality of *R*_S_ and *R*_C_ is not necessary in this case. For cancelling the effect of *R*_S_, the value of *A*_2_ is 1.5 (*V*_set_A2_ = 0.15 V) theoretically (the real value should be slightly higher, *V*_set_A2_ = 0.152 V, i.e., *A*_2_ = 1.52 when |*Z*_in_min_| = 0.2 Ω, which is a sufficiently low value). This uncertainty is given by the tolerance/dispersion of the *R*_X_ (50–65 Ω) and *R*_S_ values. The adjustability range of gain *A*_1_ was 0.1 → 10 (*V*_set_A1_ = 0.01 → 1 V). The ideal range of *L*_tun_ reaches 10 → 0.1 mH. The simulation yields a range of 8.3 → 0.11 mH, while laboratory measurements yield 12.0 → 0.11 mH. The mechanisms of real parasitic behavior are very similar to the previous case. Moreover, as was expected, the possibility to set gain *A*_2_ precisely has a substantial effect on the low-frequency features. This effect is visible in Figure 7, where the variation in *A*_2_ is captured. The simulation-based resonant peaks in Figure 6 are located at frequencies of 525 kHz (*V*_set_A1_ = 0.01 V) and 5.45 MHz (*V*_set_A1_ = 1.00 V). The measured values for these settings are 148 kHz and 1.20 MHz (1.20 MHz also in Figure 7), respectively, as a consequence of existing parasitic capacities.

It can be seen that the value of |*Z*_in_min_| is equal to 15 Ω (when *A*_2_ = 0), based on formula

(6) $$\left|Z_{\mathrm{in}\_\min}\right|≅\frac{R_{S}R_{C}+R_{S}R_{p}-R_{C}R_{p}A_{2}}{R_{p}A_{1}}.$$

The parasitic zero can be approximately found at frequency

(7) $$\omega_{z}≅\frac{R_{S}R_{C}+R_{S}R_{p}-R_{C}R_{p}A_{2}}{LR_{p}},$$

which, in our case, results in *f*_z_ ≅ 24 kHz for *A*_2_ = 0.

Simulation-based stability tests regarding transfer in feedback loops do not reveal any significant issues, including the saturation of the DC operation points of active elements or ringing and oscillations in the presented signals in application examples. Potential issues may occur for borderline control values of gain *A* when impedance reaches very high values, and some real parasitic DC offsets may cause the saturation of active element(s) because of high loop gain.

## 4. Application Examples

We have selected two typical applications where the presented solutions of adjustable impedances have beneficial utilization. The first one targets the design of a tunable single-phase oscillator of the two-point LC family. The second one is a simple linear LC filter of the band-pass response.

### 4.1. Electronically Adjustable LC Oscillator 

**Figure 8:**
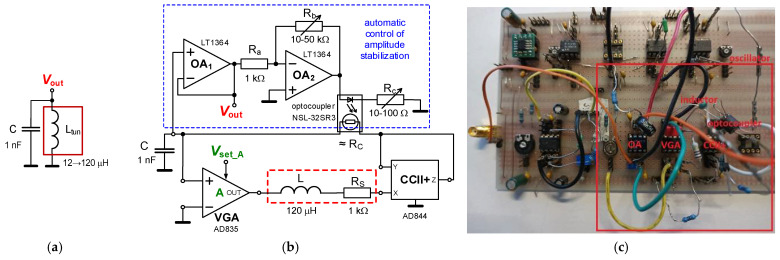
New electronically adjustable LC oscillator: (**a**) simplified ideal principle, (**b**) full topology including AGC, (**c**) PCB for experimental measurement (a universal board including adjustable inductor).

Figure 8 shows the topology of the oscillator using tunable inductance (and values of parameters, discussed in Section 3), as well as the test PCB used for experimental verification of the introduced application examples. We selected a universal printed circuit board (PCB) for verification and experimentation. It has very small areas of soldered paths (only the supply path is distributed as a PCB wire) and air-wire (including jumpers) interconnections of nodes. It must be noted that the precise design of the PCB in all tested cases is not necessary in the case of methodology verification and experimentation. The most visible limitation is the frequency of the resonant peak of impedance, approximately determined by Equation (4). Decreasing the PCB capacity shifts this peak to higher frequencies (unfortunately, the shift is insignificant). Therefore, this step was omitted. Moreover, the chosen applications (e.g., oscillator, filter) assume working capacities around 1 nF, which makes these real non-idealities (100 times smaller) insignificant.

A single additional capacitor *C* = 1 nF creates a fully operating oscillator from the circuit in Figure 2. The characteristic equation of this circuit was obtained in the form of

(8) $$s^{2}+\frac{\left(R_{S}-R_{C}\right)}{L}s+\frac{A-1}{LC}=0,$$

and gives a very simple condition of oscillation *R*_S_ ≤ *R*_C_ and an equation for oscillation frequency:

(9) $$\omega_{0}=\sqrt{\frac{1}{L_{\mathrm{tun}}C}}=\sqrt{\frac{A-1}{LC}}≅\sqrt{\frac{V_{\mathrm{set}\_A}\left(\frac{R_{f}+R_{g}}{R_{f}}\right)-1}{LC}}.$$

The relative sensitivities of the oscillation frequency to the parameters in Equation (9) reach typical values, e.g., −0.5 (because of the square root in the denominator). When we consider the middle form of (9) including gain *A*, then *S*_R_ω0_^A^ = ∂*ω*_0_/∂*A* × *A*/*ω*_0_ = *A*/(2 × (*A* − 1)). An almost identical form is valid also for *V*_set_A_ because resistors have a constant value.

**Figure 9:**
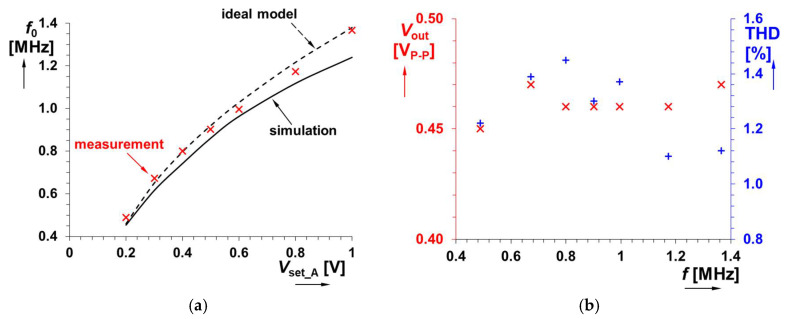
Analysis of the oscillator from Figure 8: (**a**) oscillation frequency versus driving voltage *V*_set_A1_ and (**b**) output level and THD versus oscillation frequency.

**Figure 10:**
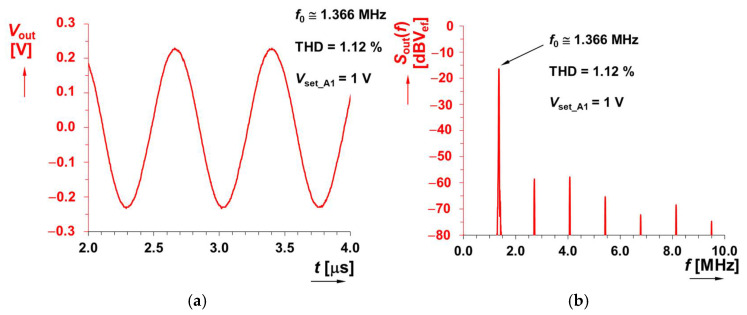
Example of output waveform for *V*_set_A1_ = 1 V (*f*_0_ = 1.366 MHz): (**a**) time domain and (**b**) frequency domain.

The value of *L* was selected as 120 μH and *R*_S_ was intentionally increased to 1 kΩ. These values are expected for the theoretical adjustability of the range of oscillation frequency *f*_0_ from 0.5 up to 1.2 MHz by changing the value of gain *A* of VGA. An example of hundreds of kHz was selected intentionally in order to show the effectivity of the method in the context of a very large *R*_S_ (a high value in comparison to RF coils that have approximately one-hundred-times lower values of *R*_S_ and are more suitable above tens of MHz) and an *L* value fitting for designs in hundreds of kHz. The theoretical design expects gain *A*_1_ in the range of 2 up to 8 (*V*_set_A1_ = 0.2 → 0.8 V) for the indicated tunability. Accurate theoretical adjustment of *f*_0_ is allowed between 0.46 and 1.22 MHz in this *V*_set_A1_ range. The PSpice simulations show that a slight increase of *V*_set_A1_ > 0.8 V for the high-frequency limit will be required; therefore, *V*_set_A1_ = 0.2 → 1 V is considered. The comparison of the theoretical, simulated and measured results for the adjustment of *V*_set_A1_ is shown in Figure 9a. Results from simulations yield a readjustment of *f*_0_ from 0.45 up to 1.24 MHz, whereas results from measurements show tunability from 0.49 up to 1.366 MHz. The output level of the oscillator reaches an approximately constant value around 0.45 V_P-P_, while the total harmonic distortion (THD) remains between 1 and 1.5 % (see Figure 9b). Figure 10 illustrates an example of an output waveform and FFT spectrum for *V*_set_A1_ = 1 V (*f*_0_ = 1.366 MHz).

The equivalence of *R*_S_ = *R*_C_ and the oscillation condition (*R*_S_ ≤ *R*_C_), respectively, are directly used for amplitude stabilization purposes. Resistor *R*_C_ was replaced by the NSL-32SR3 optocoupler [35], which decreases the value of resistance when the diode current increases, and the range of resistivity adjustment reaches values from tens of kΩ down to low hundreds of Ω [36].

The amplitude stabilization requires a buffered output voltage and its amplification, as is shown in Figure 8 (blue rectangle with dashed lines). Any rectification, a long time constant and a complex regulation loop [36] are not required because the optocoupler itself has a very slow reaction. Then, negative feedback for automatic stabilization is ensured. In the case of tunable inductance, the cancellation procedure of *R*_S_ by *R*_C_ directly includes also the possibility to set the oscillation condition (amplitude stabilization). No additional parallel negative resistor is required. Consequently, the circuitry of this electronically tunable (and amplitude stabilized) LC oscillator is very simple in comparison to many similar solutions [30].

The presented settings in Figure 10 represent a tradeoff between the tunability range, acceptable THD and usable amplitude levels. In RF systems, a much higher THD is still acceptable. In these cases, the output level could be close to 2V_p-p_ with the same supply voltage. Another possibility to increase the output voltage swing is to change the type of VGA or use a VGA with a higher supply voltage, but the bandwidth would be significantly reduced (AD835 used as VGA has the best frequency features from commercially available variable gain amplifiers). The THD would not be impacted significantly; however, power consumption would increase rapidly. Therefore, our solution represents an acceptable tradeoff of useful features and availability.

We tried to compare typical narrow-band solutions of LC oscillators intended for high-frequency purposes with our design. The proposed solution seems to be slightly complex but the intended application bandwidths (kHz, MHz) do not allow us to implement the method used in the published solutions (see Table 2 and circuits in [37,38,39,40,41,42,43,44,45]). This is because of the values and physical realization of integrated inductors (unsuitable for low frequencies due to values in nH and also their implementation). Moreover, the range of tunability allowed in these solutions is very narrow (ratio between *f*_max_ and *f*_min_ is typically around *f*_max_/*f*_min_ = 1.2, i.e., varactors allow adjustment between 1.7 and 1.9 GHz, for example). Wider tunability (e.g., from 3 up to 5 GHz) supposes the switching of LC banks (“LC tanks”) by additional control logic. The driving voltage range seems to be also very large [38,42], while the gained frequency ranges of tunability are very low. On the other hand, these systems do not need precise amplitude stabilization and low waveform purity due to different application purposes (communications) and have also very good phase noise (>80 dBc/Hz) in comparison with low-frequency solutions (typically between 40 and 80 dBc/Hz). Wideband applications at kHz and MHz require larger frequency tunability/adjustability and amplitude stability than standardly available in high-frequency LC approaches. To the best of the authors’ knowledge, low-frequency LC oscillators have not been designed frequently yet, because synthetic equivalents of inductances [45] offer inductor-less operation. However, manufacturers have offered also some low-quality (high serial losses) inductors in a small applicable package for several years. Therefore, it is worth considering their implementation (electronic tunability and elimination of losses) in common designs, which is also purpose of this work.

The general LC oscillator topology or the Colpitts one [37,45] can be considered simpler than the topology of the oscillator presented in this paper. However, our topology is complete and it also includes a biasing and amplitude stabilization system. Moreover, the standard LC oscillators (including the Colpitts one) do not allow wideband electronic tunability (by driving DC voltage) because the capacity of varicaps/varactors (standardly used in LC oscillators) has a very limited adjustment range. The electronic tunability of the oscillation frequency by the inductance value (as presented in our case) has not been tested in recently published works because it has certain limitations, especially at high frequencies (extensive active circuitries), but can be used without issues in kHz and MHz bands with significant advantages (wideband tuning). The wideband tunability of the frequency with almost constant output levels and low THD requires the implementation of a circuit for amplitude stabilization, which is standardly not used in LC oscillators (including the Colpitts type) because of their narrowband tunability [37,38,39,40,41,42,43,44,45].

### 4.2. Electronically Tunable LC Band-Pass Filter 

**Figure 11:**
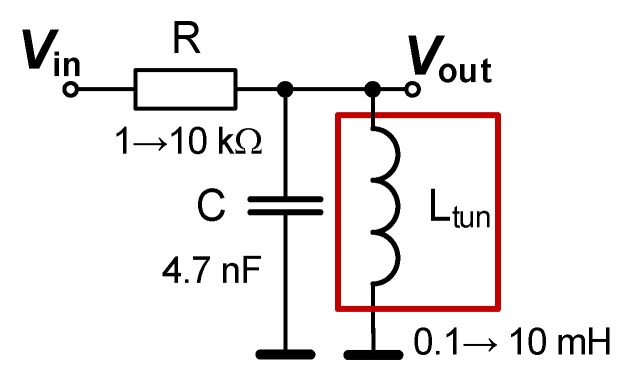
Electronically tunable LC band-pass filter.

Cancellation of the serial resistance of the real coil is beneficial for many linear applications (active filters especially). The band-pass (BP) filter topology (see Figure 11) of this application results from a standard RLC passive equivalent [3,4] of the filter but with significant advantages. The tunable inductance from Figure 3 (electronic adjustment of *L*_tun_ and also active electronic cancellation of losses) offers one-decade tuning of the center frequency through two decades of the available range of the value of *L*_tun_ (0.1 → 10 mH). Precise minimization of the discussed parasitic effects connected with serial resistance (proper adjustment of *A*_2_) causes good attenuation in the low-frequency stop band. The resulting transfer function (then expanded to a complete form based on the parameters of the active tunable inductance solution) has the following form:

(10) $$\begin{array}{l} K_{BP}(s)=\frac{\frac{1}{RC}s}{s^{2}+\frac{1}{RC}s+\frac{1}{L_{\mathrm{tun}}C}}=\\ \frac{\frac{1}{RC}s+\frac{R_{S}-R_{C}A_{2}}{RLC}}{s^{2}+\frac{CR\left(R_{S}-R_{C}A_{2}\right)+L}{RLC}s+\frac{A_{1}R+R_{S}-R_{C}A_{2}}{RLC}} \end{array}$$

Formula (10) indicates the presence of a low-frequency parasitic zero (*f*_z_BP_ = (*R*_S_ − *R*_C_*A*_2_)/(2π*L*) = *R*_S_/(2π*L*)) and finite attenuation *K*_min_ (*ω* → 0) = *R*_S_/(*R*_S_ + *A*_1_*R*) when the *R*_S_ parameter is not cancelled by *A*_2_. Considering *A*_2_ = *R*_S_/*R*_C_ (expected lossless operation), the center frequency has the form of

(11) $$\omega_{C}=\sqrt{\frac{1}{L_{\mathrm{tun}}C}}=\sqrt{\frac{A_{1}}{LC}}≅\sqrt{\frac{V_{\mathrm{set}\_A1}\left(\frac{R_{f}+R_{g}}{R_{f}}\right)}{LC}},$$

and the bandwidth (independently of the center frequency, settable by the value of *R*) can be found as *BW* = 1/(2π⋅*RC*). The quality factor can be influenced by the value of resistor *R* (*Q* = *R*·√(*C*/*L*)). The relative sensitivities of the center frequency to important parameters (*A*_1_, *L*, *C*) are very similar to the previous oscillator (in fact, the form is identical) and it reaches −0.5 for *L* and *C* and *S*_R_ωC_^A^ = ∂*ω*_C_/∂*A*_1_ × *A*_1_/*ω*_C_ = *A*_1_/(2 × (*A*_1_ − 1)) for gain *A*_1_. The relative sensitivity of the quality factor to the value of *R* reaches 1 (+0.5 and −0.5 for *C* and *L*). These, as well as previously noted values of sensitivities, are typical for similar solutions.

**Figure 12:**
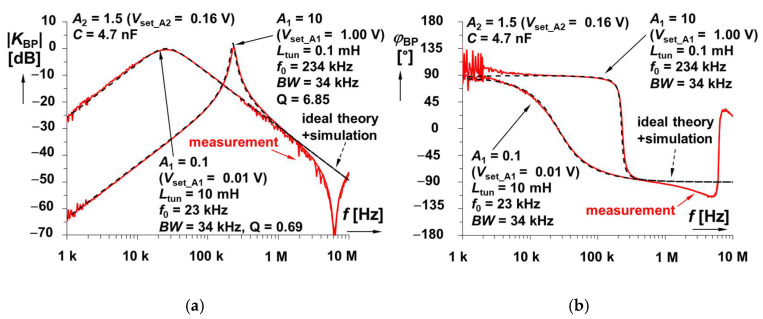
Frequency responses of electronically tunable BP LC filter: (**a**) magnitude responses and (**b**) phase responses.

When the ideal design supposes the operationability of the BP having a large value of inductance (units of mH), then operational frequencies of tens–hundreds of kHz are expected. Selection of *C* = 4.7 nF, *BW* = 35 kHz returns the value of *R* equal to 967 Ω (close to 1 kΩ value from fabrication series). The ideal tunability of center frequency *f*_C_ gives the range 23 → 234 kHz for *L*_tun_ adjustment 0.1 → 10 mH (*V*_set_A1_ = 0.01 →1 V), as used in the analysis for the topology presented in Figure 3. Further parameters are also identical to the parameters used in the design (*R*_S_ = 150 Ω, *R*_C_ = 100 Ω, *A*_2_ =1.52) discussed in Section 3.2. The simulation and measurement results show *f*_C_ between 26 and 224 kHz and 25.7 → 234 kHz, respectively. Resulting frequency responses are shown in Figure 12. It is visible that the simulated bandwidths are almost identical to the ideal cases (in fact, ideal and simulated traces are overlapping with each other), especially at the low-frequency border. In these cases, the input amplitude of the DSO-X 3024T generator (in FRA mode) was set to 200–500 mV.

## 5. Importance of the Proposed Method for Sensing, Instrumentation and Measurement

Inductors are extremely important in the fields of measurement, instrumentation and communication [46]. Moreover, these electrical components, for instance, are critical in sensing systems for electrodynamic velocity [47], energy harvesting [48], etc. The serial resistance of the inductor, representing an important real parameter of the coil, creates issues especially in the low-frequency bands (below 1 kHz) of the coil operation. Researchers in [49] clearly explain the importance of this parameter for the efficiency of near-field wireless power transfer but, depending on the frequency, it also plays a significant role in low-power sensing devices. Similarly as in [49], the serial resistance of the inductor decreases the efficiency of a receiving antenna (e.g., used in a sensor of an electromagnetic field). Commercially fabricated coils (and various types of inductors) have always this feature, influencing more or less the real operation of a system. Therefore, when a highly precise device is designed, a methodology allowing the adjustment or even cancellation of losses due to the serial resistance of the inductor is appreciated. The method introduced in this work shows how to move the features of the inductor/coil closer to an ideal device operating with an un-damped response in resonators (as documented in our experiments).

## 6. Conclusions

In this paper, a novel method of electronically adjustable inductance including the cancellation of losses (a significant undesired property of standard passive elements) was introduced. The value of adjustment has been targeted to one or two decades of tunable inductance variation from hundreds of μH up to tens of mH (in our case, from 0.1 mH up to 10 mH). The two proposed solutions have two types of adjustability of the tunable inductance value (proportional to *A* − 1 or *A*) and two methods of serial resistance cancellation (passive value and electronic adjustment) that can be used beneficially in various applications. Their experimental verification confirmed their operationability in the intended value ranges from 0.11 up to 0.95 mH and from 0.11 up to 12 mH by driving voltage *V*_set_A_ adjusted between 0.2 (or 0.01 V, respectively) up to 1 V. The measured power consumptions correspond to discrete solutions (113 and 162 mW for solutions in Figure 2 and Figure 3).

Two applications were selected for the explanation of the usefulness of the proposed method. The first application example shows the design of an LC-type oscillator offering electronic tuning and a simple solution of amplitude stabilization (direct engagement of the optocoupler for oscillation condition control). It was experimentally tested from 0.49 up to 1.37 MHz with THD below 1.5%. The second application is an RLC band-pass filter allowing a beneficial tunability range within one decade (not typical for the standard form of expression for the center frequency in the RLC solution) by a wide range of inductance adjustment. The center frequency adjustment was verified in a range from 26 kHz up to 234 kHz.

The presented approach opens up the possibility to use various materials for coil fabrication having significantly higher specific material resistivity because, as was shown, the used technique allows the cancellation of this parasitic feature. The presented methodology can be beneficially used for on-chip design (low-value inductors having losses in tens of Ω), where serial resistance issues can be very significant. This results in the possibility of the simplification or cost reduction of the inductance fabrication process, and it brings electronic adjustability of the inductance. The possibility to eliminate serial resistance precisely, as well as adjusting the value of inductance to an exact value electronically, is very beneficial because the standard tolerances of these elements are typically ±20% or even more.

The presented method of implementation and verification is not optimized for low-power design. A key contribution is in circuit theory, regardless of the power consumption of an example application. Therefore, standard off-the-shelf active elements were used. Therefore, the experimental results as well as the selected means of verification are sufficient to confirm the operationability of the proposed circuits precisely. Of course, frequency bands, as well as power consumption and voltage levels, can be optimized when required for a particular application. Power consumption could be decreased without changing the topology. In the case of having CMOS-integrated circuits replacing the functions of AD844 and AD835, optimized for low-power design, the power consumption could be up to tens of mW (based on the selected process) when similar performance is expected. Note that the inductance will be still connected to the chip externally as a discrete part (values in μH and mH are not integrable). Therefore, we selected the complete employment of discrete elements (also active parts) in our verifications of the methodology.

The active elements in the proposed topology can be constructed by the CMOS structures presented in [50] (while the forms and values of passive elements remain unchanged). However, the limited bandwidth (in comparison with features of high-power-consuming bipolar AD835) and parasitic effects would result in lower-frequency positions of the parasitic resonant peaks of the impedance plot and reduced dynamics and adjustability.

## Figures and Tables

**Table 1:** Brief comparison of typical solutions of electronically adjustable inductance simulators (impedance inverters using capacitors) with presented methodology of real inductance adjustment.

Solution	Use of Real Inductor	Number of Active/Passive Elements (Including Serial Resistance)	Driving Force	Controlled Parameter	Reported as Lossless	Cancellation of Losses (in Lossy Operation) Possible	Electronic Cancellation of Losses	Power Consumption *	FOM
[11]	No	2/1(0)	DC current	*g*_m_	N/A	No	No	N/A	0.33
[12]	No	2/3(0)	DC current	*g*_m_	N/A	No	No	up 41 mW	0.20
[13]	No	1/2(0)	DC current	*g*_m_	partially ^a^	No	No	0.9 mW	0.33
[14]	No	1/2(0)	-	*g*_m_	Yes	No	No	N/A	0.33
[15]	No	1/2(0)	DC current	*g*_m_	Yes	No	No	N/A	0.33
[16]	No	1/2(0)	DC current	*g*_m_	Yes	No	No	N/A	0.33
[17]	No	4/4(0)	DC voltage	*B*	No	Yes	Yes	N/A	0.38
[18]	No	4/3(0)	DC voltage	*A*	Yes	No	No	N/A	0.14
[19]	No	1/2–3(0)	DC current	*g*_m_	partially ^a^	No	No	N/A	0.33
[20]	No	1/2(0)	DC current	*g*_m_	No	No	No	5.7 mW	0.33
[21]	No	1/2(0)	DC current	*g*_m_	N/A	No	No	N/A	0.33
[22]	No	1/2(0)	DC current	*g*_m_	No	No	No	63 mW	0.33
[23]	No	2/2(0)	DC current	*g*_m_	No	No	No	N/A	0.25
[24]	No	2/1–2(0)	DC current	*g*_m_	partially ^a^	No	No	N/A	0.33
[25]	No	2/2(0)	DC voltage	*g*_m_	Yes	No	No	20 mW	0.25
Figure 2	Yes	2/2(3)	DC voltage	*A*	Yes	Yes	No	113 mW	0.50
Figure 3	Yes	3/2(3)	DC voltage	*A*	Yes	Yes	Yes	162 mW	0.60

N/A—not available, ^a^ partially—some reported solutions have this capability, * very low power consumptions are valid for solutions using low-voltage CMOS technologies, *g*_m_—transconductance adjustment, *B*—current gain adjustment, *A*—voltage gain adjustment, FOM = (electronic control + cancelation of losses + electronic control of losses)/(number of active + passive elements without losses) where, when particular feature is available, the numerator equals 1; if not, 0 is inserted.

**Table 2:** Brief comparison of typical LC oscillators and proposed type.

Solution	Colpitts Type	Number and Type of Active/Passive Elements (Including Serial Resistance and Amplitude Stabilization)	Bias Point Setting Not Required	Continuous Electronic Tuning Allowed (in Full Range)	Switching of Capacitor/Inductor Banks Not Required for Full Range	Driving Force	Amplitude Stabilization (Almost Constant Output Level at All Frequencies)	Tunability Range Ratio (*f*_max_/*f*_min_ in Single Band without Switching)	Application Bands	Suitable for Low Frequency Bands
[37]	Yes	1 BJT/4	No	N/A	N/A	N/A	N/A (N/A)	N/A	N/A	N/A
[38]	No	2 CMOS/8+	No	Yes (No)	No	Voltage (0.4 → 2.3 V)	No (N/A)	≅1.3–1.5	GHz	No
[39]	No	5+ CMOS/3+	No	Yes (No)	No	Voltage (0 → 1.2 V)	No (N/A)	≅1.02	GHz	No
[40]	No	5+ CMOS/5+	No	Yes (No)	No	Voltage (0 → 1.2 V)	N/A (N/A)	≅1.02	GHz	No
[41]	No	5+ CMOS/5+	No	Yes (No)	No	Voltage (0 → 1.8 V)	N/A (N/A)	≅1.06	GHz	No
[42]	No	1–2 BJT, 2 CMOS/8+	No	Yes (No)	No	Voltage (0 → 3.0 V)	No (N/A)	≅1.2	GHz	No
[43]	No	4 CMOS/5	Yes	Yes (No)	Yes	Voltage (0 → 0.9 V)	N/A (Yes)	≅1.1	GHz	No
[44]	Yes	1 amplifier, 2+ CMOS/3 or more	No	N/A	N/A	N/A	N/A (N/A)	N/A	N/A	N/A
[45]	No	2 CMOS/3	No	No	No	Voltage (0.7 → 1.2 V	N/A (N/A)	≅1.2	GHz	No
Figure 8	No	2 OA, VGA, CCII/6	Yes	Yes (Yes)	Yes	Voltage (0.2 → 1 V)	Yes	≅2.8	kHz, MHz	Yes

+ additional banks (transistors for switching and passive elements) can be added based on full required range, N/A—not available.

## Data Availability

Not applicable.
